# Cement augmentation of humeral head screws reduces early implant-related complications after locked plating of proximal humeral fractures

**DOI:** 10.1007/s11678-018-0440-x

**Published:** 2018-01-30

**Authors:** J. Christoph Katthagen, O. Lutz, C. Voigt, H. Lill, A. Ellwein

**Affiliations:** 1 Department of Orthopaedic and Trauma Surgery, DIAKOVERE Friederikenstift, Hannover, Germany; 2 Department of Trauma, Hand and Reconstructive Surgery, Universitätsklinikum Münster, Albert-Schweitzer-Str. 1, 48149 Münster, Germany

**Keywords:** Humeral fractures, proximal, Shoulder, Bone plates, Cementation, Postoperative complications, Humerusfrakturen, proximale, Schulter, Knochenplatten, Zementierung, Postoperative Komplikationen

## Abstract

**Background:**

Cement augmentation (CA) of humeral head screws in locked plating of proximal humeral fractures (PHF) was found to be biomechanically beneficial. However, clinical outcomes of this treatment have not been well evaluated to date.

**Objectives:**

To assess outcomes of locked plating of PHF with additional CA and to compare them with outcomes of conventional locked plating without CA.

**Methods:**

24 patients (mean age, 74.2 ± 10.1 years; 22 female) with displaced PHF were prospectively enrolled and treated with locked plating and additional CA. The Constant score (CS), the Simple Shoulder Test (SST), and the Simple Shoulder Value (SSV) were assessed 3 and 12 months postoperatively. Fracture healing and potential complications were evaluated on postoperative radiographs. The CS and complications were compared with the outcomes of a matched group of 24 patients (mean age, 73.9 ± 9.4 years; 22 female) with locked plating of displaced PHF without CA.

**Results:**

At the 3‑month follow-up, the mean CS was 59.9 ± 15.6 points, the mean SST was 7.5 ± 2.7 points, and the mean SSV was 63.9 ± 21.7%. All scores significantly improved by the 12-month follow-up (*p* < 0.05; CS, 72.9 ± 17.7; SST, 9.2 ± 3.2; SSV, 77.2 ± 17.3%). There were two cases (8%) of biological complications (*n* = 1 varus malunion and *n* = 1 humeral head necrosis). Compared with locked plating without CA, no significant differences were observed between the CS at the 3‑ (57.8 ± 13.4 points; *p* = 0.62) and 12-month (73.0 ± 12.8 points; *p* = 0.99) follow-up. However, patients without CA had a significantly increased risk of early loss of reduction and articular screw perforation (*p* = 0.037).

**Conclusion:**

Locked plating of proximal humeral fractures with trauma cement augmentation of humeral head screws could be translated from the ex-vivo lab setting into the clinical situation without additional complications. Locked plating of displaced PHF with additional cement augmentation showed similar clinical outcomes but reduced the rate of early implant-related complications compared to locked plating without additional CA.

## Zusammenfassung

**Hintergrund:**

Biomechanisch begünstigt die additive Zementaugmentation (ZA) von Humeruskopfschrauben bei winkelstabiler Plattenosteosynthese proximaler Humerusfrakturen (PHF) die Stabilität und Versagenslast. Bislang sind klinische Ergebnisse dieser Versorgungsform weitgehend unbekannt.

**Ziel der Arbeit:**

Die Ergebnisse nach winkelstabiler Plattenosteosynthese dislozierter PHF mit ZA sollten evaluiert und mit jenen ohne ZA verglichen werden.

**Methoden:**

In die Studie wurden 24 Patienten (durchschnittlich 74,2 ± 10,1 Jahre; 22 w.) mit dislozierten PHF prospektiv eingeschlossen und mittels winkelstabiler Plattenosteosynthese mit additiver ZA operativ behandelt. Nach 3 und 12 Monaten wurden der Constant Score (CS), der Simple Shoulder Test (SST) sowie der Simple Shoulder Value (SSV) erhoben. Anhand von Röntgenbildern wurden die knöcherne Konsolidierung sowie etwaige Komplikationen evaluiert. Der CS und die Komplikationsrate wurden mit den Ergebnissen eines Matched-Pair-Kollektivs von 24 Patienten (73,9 ± 9,4 Jahre; 22 w.) mit winkelstabiler Plattenosteosynthese dislozierter PHF ohne ZA verglichen.

**Ergebnisse:**

Nach 3 Monaten betrug der CS durchschnittlich 59,9 ± 15,6 Punkte, der SST lag bei 7,5 ± 2,7 Punkten und der SSV bei 63,9 ± 21,7 %. Sämtliche Scores verbesserten sich zum 12-Monats-Zeitpunkt signifikant (*p* < 0,05; CS 72,9 ± 17,7 Punkte, SST 9,2 ± 3,2 Punkte; SSV 77,2 ± 17,3 %). Bei 2 Patientinnen (8 %) wurden biologische Komplikationen beobachtet (*n* = 1 Varuspseudarthrose und *n* = 1 Humeruskopfnekrose). Beim Vergleich mit dem CS der Patienten mit winkelstabiler Plattenosteosynthese ohne ZA zeigten sich keine signifikanten Unterschiede 3 Monate (57,8 ± 13,4 Punkte; *p* = 0,62) sowie 12 Monate (73,0 ± 12,8 Punkte; *p* = 0,99) postoperativ. Allerdings zeigte sich bei Patienten mit konventioneller Plattenosteosynthese ohne ZA ein signifikant höheres Risiko für einen Repositionsverlust mit intraartikulärer Schraubenperforation (*p* = 0,037).

**Schlussfolgerung:**

Die ZA von Humeruskopfschrauben bei Plattenosteosynthese dislozierer PHF konnte ohne zusätzliche Komplikationen aus dem biomechanischen Labor in die klinische Anwendung übertragen werden. Mit der additiven ZA bei winkelstabiler Plattenosteosynthese von PHF war die Rate an implantatassoziierten Komplikationen im Vergleich zur winkelstabilen Plattenosteosynthese ohne ZA bei vergleichbaren klinischen Ergebnissen reduziert.

## Introduction

Locked plating of displaced proximal humeral fractures (PHF) poses a serious challenge to orthopedic trauma surgeons. The biologic circumstances of the mainly older patient population with osteoporotic bone stock make screw anchorage difficult [1–3]. The unique mechanical setting with the humeral head articulating surface being loaded against the screw tips and the rotator cuff pulling in an antero- and posteromedial direction demands for a fracture fixation with immediate stability [4, 5]. However, the ideal implant characteristics should make the construct flexible enough to unload the bone implant interface but rigid enough to minimize fracture movements [6].

Despite identification of several potential risk factors for fixation failure and regardless of the continuous development of implants with improved plate and screw design, the complication rate associated with locked plating of PHF is still high [2, 7, 8]. Especially in fragility fractures of the older population, loss of reduction with secondary articular screw perforation and varus displacement with loss of fixation can still be observed in up to 20% of cases within the first 12 months postoperatively [9, 10].

Humeral head screw augmentation with polymethyl methacrylate (PMMA) trauma cement has been shown to enhance screw anchorage and to thereby increase the primary stability of locked plating of displaced PHF in several biomechanical studies [11–14]. The primary goal of this study was to evaluate whether these in vitro findings can be translated into daily clinical routine without additional complications compared with conventional locked plating without additional trauma cement augmentation. The secondary goal was to compare functional outcomes and complication rates between locked plating of PHF with cement augmentation versus without cement augmentation of humeral head screws. We hypothesized that locked plating of PHF with additional cement augmentation of humeral head screws can be used in clinical practice without additional complications and that early implant-related complications can be reduced compared with locked plating without additional cement augmentation of humeral head screws.

## Methods

Between February 2014 and September 2015, 39 patients underwent fracture fixation of a displaced PHF with the PHILOS plate (DePuy Synthes®, Umkirch, Germany) along with additional humeral head screw augmentation with PMMA trauma cement in this prospective case series. The study was approved by the institutional review board and informed consent was obtained from all individual participants included in the study. Inclusion criteria were:

1. Displaced unilateral PHF
2. Age of at least 18 years
3. Written informed consent

Patients were excluded from the study if they:

4. Had a bilateral PHF and/or
5. Had previous shoulder surgery or a neurologic disease with impairment of the upper extremity and/or
6. Refused to participate

Preoperative patient-specific data such as age, gender, affected side, preexisting diseases and medications, as well as concomitant fractures were noted. The PHF were preoperatively classified according the Codman segment theory and the classification suggested by Resch et al. [15, 16]. The neck–shaft angle (NSA) was measured on a standardized true anteroposterior (AP) view on the second day after surgery [17]. X‑rays were further analyzed for primary screw perforation, for displaced fragments, and for the quality of reduction according to Marc Schnetzke et al. [8, 18]. Failure was defined as a “malreduced” situation according to Schnetzke et al. [8].

### Titanium plating with cement augmentation of humeral head screws

The PHILOS plate (DePuy Synthes®) is made of titanium alloy. The shaft was fixed with three 3.5-mm screws in all cases. The number of 3.5-mm humeral head screws used ranged from 5 to 9 (on average, 7). An average of three cannulated humeral head screws (range, 2–5) were augmented with 0.5–1 ml of trauma cement (PMMA). The lengths of the humeral head screws were selected so that their tips extended to the subchondral surface of the humeral head without penetration of the articular surface. The surgeries were performed according to the manufacturer’s instructions and guidelines via the deltopectoral approach by five different orthopedic surgeons, all familiar with PHF treatment. If the fracture involved the bicipial groove, an epiosseous soft-tissue tenodesis of the long head of the biceps tendon was performed. The arm was put in a sling for comfort only for the first week postoperatively. Free passive and active range of motion was allowed immediately after surgery without weight-bearing for 6 weeks.

### Functional and radiological follow-up

Patients were followed up clinically and radiologically at 3 months postoperatively and again clinically at 12 months postoperatively. At the 3‑month follow-up, patients had radiographs in two planes (true AP and axial) unless previous radiographs had demonstrated fracture consolidation. The NSA was measured again [17]. Furthermore, both radiographic planes were evaluated for:

1. Screw perforation
2. Loss of fixation
3. Fragment displacement
4. Bony fracture consolidation
5. Avascular necrosis of the humeral head and/or the tuberosities
6. Implant loosening.

The clinical examination at the 3‑month and 12-month follow-up included assessment of the active range of shoulder motion, the Constant score as well as the age- and gender adjusted Constant score of both the affected and nonaffected shoulders [19]. In addition, the Simple Shoulder Test (SST) [20], the Simple Shoulder Value (SSV) [21], and the average pain measured with the Visual Analog Scale (VAS) of the affected shoulder were assessed. Furthermore, all complications and reoperations were evaluated and noted.

### Patients with conventional titanium locked plating

A case-control cohort of 24 patients (two male, 22 female; mean age, 73.9 ± 9.4 years) treated with a conventional titanium locking plate was used to compare the Constant score and the complication rates with the results of the patients treated with additional cement augmentation of humeral head screws. These 24 patients derived from a historic retrospective cohort of 74 patients (54 females, 20 males; mean age, 73 years) treated with the PHILOS PHF plate. The best match with regard to gender, age, affected side, and fracture complexity according to Codman’s segment theory was sought. The investigator who matched 24 patients of the historic PHILOS group without additional cement augmentation was blinded to functional outcomes, complications, and reoperations. Apart from cement augmentation of humeral head screws, the operative technique and the postoperative rehabilitation protocol were identical to those of patients with locked plating and additional cement augmentation.

### Statistical analysis

All data was analyzed by means of descriptive statistics. Normal distribution was tested with the Kolmogorov–Smirnoff test and could not be assumed. The results of two different follow-up time points within the same group were compared with the use of the Wilcoxon matched-pair test. The results of the different groups (augmented PHILOS versus conventional PHILOS) were compared with the Mann–Whitney *U *test. The significance level was set at *p* < 0.05. The chi-square test was used to investigate any associations between the type of fixation used and the likelihood of early loss of reduction and screw perforation within the first 6 months postoperative. To overcome grouping bias, the likelihood for early loss of reduction and articular screw perforation was additionally compared for the augmented PHILOS group versus the entire historic cohort of 74 patients with use of the chi-square test.

## Results

Ten patients were excluded from the study: two patients died before final follow-up due to non-fracture-related comorbidities, six patients refused to participate, and two patients had preexisting neurologic diseases with impairment of the upper extremity. Of the remaining 29 patients, five were lost to follow-up and 24 patients (22 female, two male) with a mean age of 74.2 ± 10.3 years (range, 52–96 years) participated in final follow-up (83%).

All 24 patients (100%) were available for the 3‑month follow-up as well as for the 12-month examination. Three of the 24 patients had a two-part fracture, 17 patients had a three-part, and four patients had a four-part fracture according to the Codman segment theory [15]. The fractures were classified as varus-displaced in 14 and as valgus-displaced fractures in ten cases according to Resch et al. [16]. The NSA averaged 135.3 ± 6.3° on the second postoperative day and no primary screw perforation, fragment displacement, or implant failure was seen. According to Schnetzke et al., the reduction was anatomical or acceptable in all cases [8].

### Radiological follow-up

**Fig. 1:**
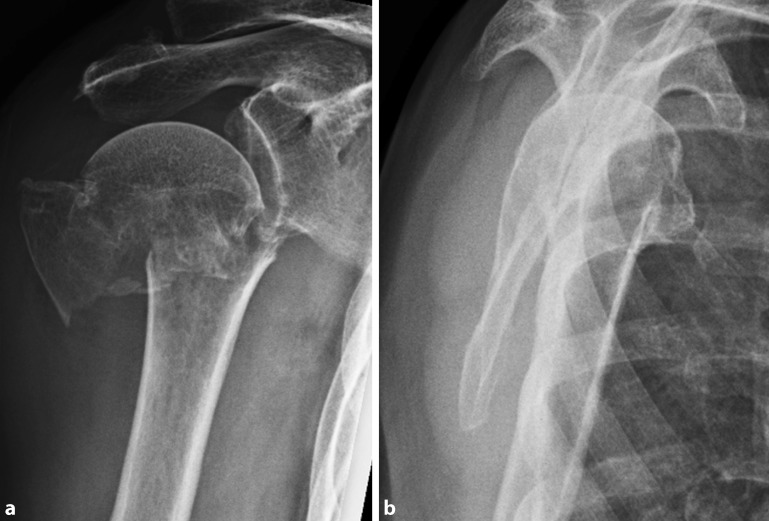
Preoperative radiographs of an 87-year-old female patient with valgus-displaced three-part proximal humeral fracture (**a** anteroposterior, **b** Y view)

**Fig. 2:**
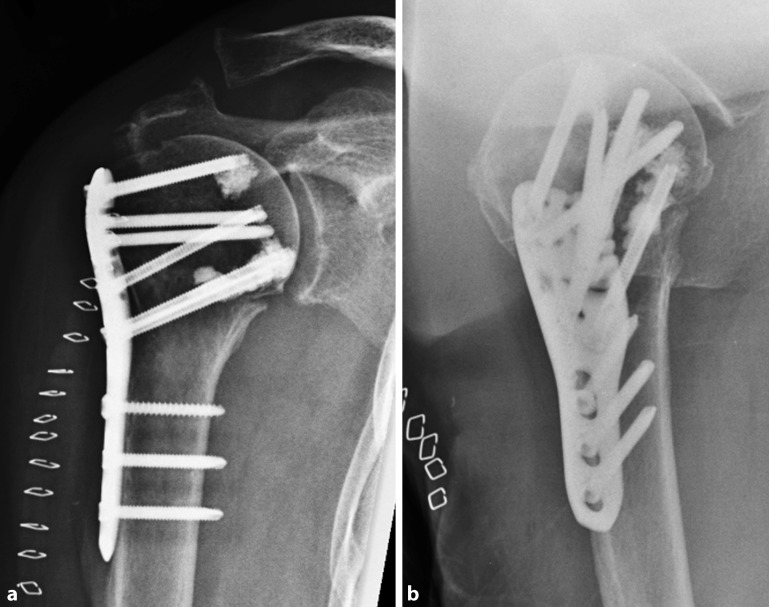
Postoperative radiographs of an 87-year-old female patient after locked plating of a proximal humeral fracture (see Fig. 1) with additional cement augmentation of the anterosuperior and inferior humeral head screws (**a** anteroposterior, **b** axillary view)

**Fig. 3:**
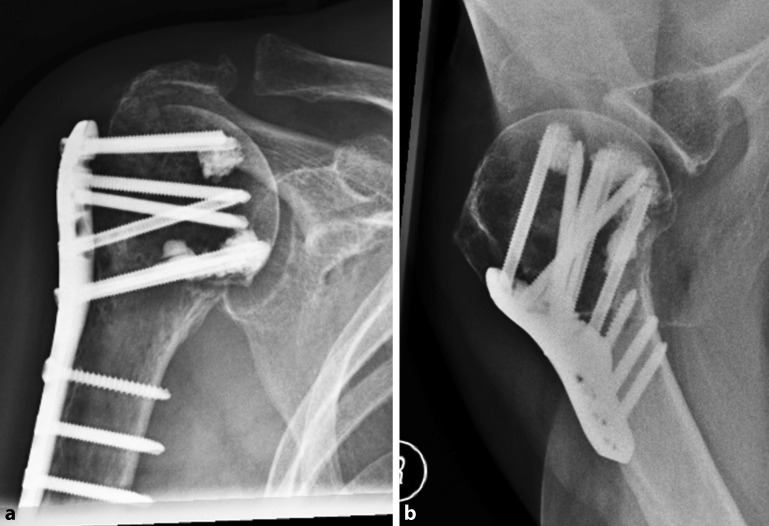
Radiographs 3 months after locked plating of a proximal humeral fracture (see Fig. 1) with additional cement augmentation of the anterosuperior and inferior humeral head screws showing fracture union without implant related complications (**a** anteroposterior, **b** axillary view)

Radiological follow-up at a mean of 3.3 months postoperatively revealed no secondary screw perforation and there was no case of secondary fragment displacement or loss of fixation (Figs. 1, 2, and 3). According to Schnetzke et al., the reduction was anatomical or acceptable in all cases without changes to a situation defined as malreduced compared with the situation on the second day after surgery. The NSA averaged 131.1 ± 8.4° at the time of the radiological follow-up, with the average loss of reduction being 4.6 ± 7.2°.

### Clinical follow-up

All functional outcomes improved from the 3‑month to the 12-month follow-up (Table 1). At 3‑months postoperatively, no neurovascular complications or infections were observed. Six out of 24 patients (25%) had an early arthroscopic revision surgery with arthrolysis, biceps tenotomy, and arthroscopically assisted implant removal between 3 and 5 months after the initial facture fixation. Intraoperative inspection of the humeral head articulating surface confirmed no articular screw perforation. The revision surgeries were performed in patients with high functional expectations but limited postoperative range of motion. At 12 months postoperatively, the average age- and gender- related Constant score was rated excellent with 100 ± 28.6%, the average SST was 9.2 ± 3.3 out of 12 possible points (Table 1). At the final 12-month follow-up, two patients (8%) had major biological complications. One patient suffered from varus nonunion due to a large defect zone in the subcapital calcar region. Another patient sustained an avascular necrosis of the humeral head with collapse of the articulating surface. No implant-related complications were observed.

**Table 1:** Clinical and functional results after PHF fixation with the PHILOS plate with additional trauma cement augmentation of humeral head screws

	VAS pain	Abduction	CS	Age- and gender-adapted CS	SST	SSV
3 months postoperatively (*n* *=* 24/24)	3.2 ± 2.7	107.3 ± 33.7°	59.9 ± 15.9 points	88.5 ± 26.7%	7.5 ± 2.8 points	63.9 ± 21.7%
12 months postoperatively (*n* *=* 24/24)	1.8 ± 2.1	127.9 ± 38.1°	72.9 ± 18.1 points	100 ± 28.6%	9.2 ± 3.3 points	77.2 ± 17.7%
*p*	0.025^a^	0.006^a^	<0.001^a^	<0.001^a^	0.01^a^	0.001^a^

*CS *Constant score*, PHF* proximal humeral fracture, *SST *Simple Shoulder Test,* VAS* visual analog scale, *SSV* Simple Shoulder Value

^a^Statistically significant difference between the 3‑ and 12-month follow-ups

### Comparison with conventional locked plating without cement augmentation

The gender of the 24 patients treated with the conventional PHILOS plate without trauma cement augmentation matched in all cases. Age differed by 1–7 years in 18 patients without significant difference between the mean age of both groups (augmented PHILOS, 74.2 ± 10.3 years; conventional PHILOS, 73.7 ± 9.7 years; *p* = 0.925). The affected side differed in four patients, one patient in the conventional PHILOS group had a less complex two-part fracture instead of a three-part fracture, and one patient in the conventional PHILOS group had a more complex four-part fracture instead of a three-part fracture compared with the cement-augmented PHILOS group. Results were available for all 24 patients (100%) in the matched conventional PHILOS cohort at the 3‑month and 12-month follow-up. No significant differences were observed between functional outcomes in both groups after 3 months and after 12 months (*p* > 0.05; Table 2). Within the first 6 months postoperatively, four patients in the conventional PHILOS group had suffered one or more screw perforations in the context of varus loss of reduction, whereas no such complication was observed within the first 6 months postoperatively in the cement-augmented PHILOS group. Patients treated with the conventional PHILOS plate without cement augmentation of humeral head screws were significantly more likely to suffer from early loss of reduction with articular screw perforations than were patients treated with cement augmentation of the humeral head screws (*p* = 0.037). The likelihood of early loss of reduction and articular screw perforation of the entire historic cohort (*n* = 19 out of 74) was also statistically significantly higher when compared with the group of patients with locked plating and additional cement augmentation (*n* = 0 out of 24; *p* = 0.006).

**Table 2:** Comparison of patient characteristics and functional outcomes in conventional PHILOS vs. cement-augmented PHILOS groups

	PHILOS with cement augmentation of humeral head screws	Conventional PHILOS without cement augmentation	*p*
CS after 3 months	59.9 ± 15.9	57.8 ± 13.7	0.853
CS after 12 months	72.9 ± 18.1	73.0 ± 13.1	0.557
CS affected side/CS nonaffected side after 12 months	82.2 ± 20.0%	84.1 ± 12.9%	0.496

*CS* Constant score

## Discussion

The most important finding of this study was that locked plating of PHF with trauma cement augmentation of humeral head screws can be translated from the ex vivo lab setting into the clinical situation without additional complications when compared with conventional locked plating. Furthermore, the additional use of cement augmentation of the humeral head screws significantly reduced the rate of early implant-related complications.

Locked plating is commonly used for the treatment of displaced PHF. However, owing to the mainly osteoporotic bone structure of the affected older patient population and the associated difficult screw anchorage, complication rates are still high. Common complications are primary and secondary screw perforation of the articular surface and varus loss of reduction. Cement augmentation by filling bony voids in the humeral head to improve the humeral head stability has been used for years; however, this procedure seems most applicable to the less common valgus-impacted PHF [22].

In 2012, Unger et al. presented the results of a cadaveric study in which the biomechanical properties of PHF fixation with conventional locked plating was compared with locked plating with additional cement augmentation of the humeral head screws [11]. The authors observed significantly more cycles to failure with cement-augmented humeral head screws compared with conventional, nonaugmented screws. Furthermore, failure load did correlate with the humeral head bone mineral density (BMD) in patients without cement augmentation, but not when cement augmentation of the humeral head screws was used. The improved stability of PHF fixation with cement augmentation of humeral head screws was confirmed by subsequent biomechanical studies. Röderer et al. showed that the screw augmentation could compensate for a low BMD, and Kathrein et al. found decreased per cycle motion and decreased varus impaction when humeral head screw tips were augmented with trauma cement [12, 13]. Since hardening of the cement is accompanied by an exothermic reaction, Blazejak et al. investigated whether heat development during augmentation may set the subchondral bone and the chondral surface at risk for necrosis and apoptosis [23]. Augmentation of the proximal screws of the PHILOS plate with PMMA led to a locally limited development of supraphysiological temperatures in the cement cloud and closely around it. The critical threshold value for necrosis and apoptosis of cartilage and subchondral bone reported in the literature, however, was not reached. Beside concerns with the thermic reaction, the question was raised of whether implants could still be removed even if cement augmentation was used. Goetzen et al. showed that screw removal was possible without any problems despite cement augmentation [24]. This was clinically confirmed in this study, since implant removal was performed without problems in six patients with cement-augmented humeral screws. In a more recent biomechanical study, Schliemann et al. reported that augmentation of anterior humeral head screws reduced the motion at the bone–implant interface [14].

The presented outcomes of this case series of patients treated with cement-augmented proximal humeral locked plating support the biomechanical findings in preceding studies. Compared with locked plating of PHF without cement augmentation of humeral head screws, significantly fewer screw perforations of the humeral head articulating surface were observed. Moreover, no additional cement-related complications were noted.

The raised awareness of concomitant glenohumeral pathologies and the knowledge of significant functional improvement after early elective arthroscopic revision surgery have led to an increased indication for this procedure, which is reflected by the 25% rate of planned, elective arthroscopies in the postoperative course [25].

Nonunion and avascular necrosis of the humeral head that were observed in two patients after locked plating with cement augmented humeral head screws are biologic complications that are related to the humeral head anatomy and blood supply and not necessarily to the type of humeral head fixation. Both types of biologic complications have been found with an incidence of up to 10% within the first 12 months postoperatively after locked plating without additional cement augmentation and should therefore be viewed as nonimplant-related complications [26, 27]. Furthermore, both types of complications have been described in the Boileau classification of so-called fracture sequelae and can be observed after operative as well as nonoperative treatment of PHF [28, 29].

### Limitations

There are several limitations that must be considered when interpreting the presented findings. First, the retrospective study design with a matched group comparison of a historic patient group has inherent bias. Second, no measurement of bone quality was available or reliably feasible in a retrospective fashion. Third, it was not evaluated whether fixation failures, on one hand, and early arthroscopic revision surgeries, on the other hand, had an impact on the final clinical outcome. Future investigations with prospective randomized comparisons of both fracture fixation techniques and with evaluation of the influence of bone quality are warranted.

## Conclusion

Locked plating of PHF with trauma cement augmentation of humeral head screws was translated from the ex vivo lab setting into the clinical situation without additional complications. Locked plating of displaced PHF with additional cement augmentation showed similar clinical outcomes but reduced the rate of early implant-related complications compared with locked plating without additional cement augmentation.

## References

[1] Singh A, Adams AL, Burchette R, Dell RM, Funahashi TT, Navarro RA (2015). The effect of osteoporosis management on proximal humeral fracture. J. Shoulder Elbow Surg..

[2] Hardeman F, Bollars P, Donnelly M, Bellemans J, Nijs S (2012). Predictive factors for functional outcome and failure in angular stable osteosynthesis of the proximal humerus. Injury.

[3] Katthagen JC, Grabowski S, Huber M, Jensen G, Voigt C, Lill H (2016). Epidemiology and treatment reality of proximal humeral fractures at a level-1 trauma center. Obere Extremität.

[4] Feerick EM, Kennedy J, Mullett H, FitzPatrick D, McGarry P (2013). Investigation of metallic and carbon fiber PEEK fracture fixation devices for three-part proximal humeral fractures. Med Eng Phys.

[5] Voigt C, Hurschler C, Rechi L, Vosshenrich R, Lill H (2009). Additive fiber-cerclages in proximal humeral fractures stabilized by locking plates: no effect on fracture stabilization and rotator cuff function in human shoulder specimens. Acta Orthop.

[6] Lill H, Hepp P, Korner J, Kassi JP, Verheyden AP, Josten C, Duda GN (2003). Proximal humeral fractures: how stiff should an implant be? A comparative mechanical study with new implants in human specimens. Arch Orthop Trauma Surg.

[7] Sproul RC, Iyengar JJ, Devcic Z, Feeley BT (2011). A systematic review of locking plate fixation of proximal humeral fractures. Injury.

[8] Schnetzke M, Bockmeyer J, Porschke F, Studier-Fischer S, Grützner PA, Guehring T (2016). Quality of reduction influences outcome after locked-plate fixation of proximal humeral type-C fractures. J Bone Joint Surg Am.

[9] Beeres FJP, Hellensleben NDL, Rhemrev SJ, Goslings JC, Oehme F, Meylaerts SAG, Babst R, Schep NWL (2017). Plate fixation of the proximal humerus: an international multicenter comparative study of postoperative complications. Arch Orthop Trauma Surg.

[10] Katthagen JC, Huber M, Grabowski S, Ellwein A, Jensen G, Lill H (2017). Failure and revision rates of proximal humeral fracture treatment with the use of a standardized treatment algorithm at a level-1 trauma center. J Orthop Traumatol.

[11] Unger S, Erhart S, Kralinger F, Blauth M, Schmoelz W (2012). The effect of in situ augmentation on implant anchorage in proximal humeral head fractures. Injury.

[12] Röderer G, Scola A, Schmoelz W, Gebhard F, Windolf M, Hofmann-Fliri L (2013). Biomechanical in vitro assessment of screw augmentation in locked plating of proximal humeral fractures. Injury.

[13] Kathrein S, Kralinger F, Blauth M, Schmoelz W (2013). Biomechanical comparison of an angular stable plate with augmented and non-augmented screws in a newly developed shoulder bench test. Clin Biomech.

[14] Schliemann B, Seifert R, Rosslenbroich SB, Theisen C, Wähnert D, Raschke MJ, Weimann A (2015). Screw augmentation reduces motion at the bone-implant interface: a biomechanical study of locking plate fixation of proximal humeral fractures. J. Shoulder Elbow Surg..

[15] Codman EA, Codman EA (1934). Fractures in relation to the subacromial bursa. The shoulder, rupture of the supraspinatus tendon and other lesions in or about the subacromial bursa.

[16] Resch H, Tauber M, Neviaser RJ, Neviaser AS, Majed A, Halsey T, Hirzinger C, Al-Yassari G, Zyto K, Moroder P (2016). Classification of proximal humeral fractures based on a pathomorphological analysis. J. Shoulder Elbow Surg..

[17] Hertel R, Knothe U, Ballmer FT (2002). Geometry of the proximal humerus and implications for prosthetic design. J. Shoulder Elbow Surg..

[18] Katthagen JC, Ellwein A, Lutz O, Voigt C, Lill H (2017). Outcomes of proximal humeral fracture fixation with locked CFR-PEEK plating. Eur J Orthop Surg Traumatol.

[19] Constant CR, Gerber C, Emery RJH, Sojbjerg JO, Gohlke F, Boileau P (2003). A review of the constant score: modifications and guidelines for its use. J. Shoulder Elbow Surg..

[20] Lippitt SB, Harryman DT, Matsen FA, Matsen FA, Fu FH, Hawkins RJ (1993). A practical tool for evaluating function: the simple shoulder test. The shoulder: a balance of mobility and stability.

[21] Fuchs B, Jost B, Gerber C (2000). Posterior-inferior capsular shift for the treatment of recurrent, voluntary posterior subluxation of the shoulder. J Bone Joint Surg Am.

[22] Robinson CM, Page RS (2003). Severely impacted valgus proximal humeral fractures. Results of operative treatment. J Bone Joint Surg Am.

[23] Blazejak M, Hofmann-Fliri L, Büchler L, Gueorguiev B, Windolf M (2013). In vitro temperature evaluation durgin cement augmentation of proximal humerus plate screw tips. Injury.

[24] Goetzen M, Windolf M, Schmoelz W (2014). Augmented screws in angular stable plating of the proximal humerus: What to do when revision is needed?. Clin Biomech.

[25] Katthagen JC, Hennecke D, Jensen G, Ellwein A, Voigt C, Lill H (2014). Arthroscopy after locked plating of proximal humeral fractures: implant removal, capsular release, and intra-articular findings. Arthroscopy.

[26] Greiner S, Kääb MJ, Haas NP, Bail HJ (2009). Humeral head necrosis rate at mid-term follow-up after open reduction and angular stable plate fixation for proximal humeral fractures. Injury.

[27] Court-Brown CM, McQueen M (2008). Nonunions of the proximal humerus: their prevalence and functional outcome. J Trauma.

[28] Boileau P, Chuinard C, Le Huec JC, Walch G, Trojani C (2006). Proximal humerus fracture sequelae: impact of a new radiographic classification on arthroplasty. Clin Orthop Relat Res.

[29] Lill H, Voigt C, Jensen G, Warnhoff M, Katthagen JC (2015). Corrective osteosynthesis of proximal humeral fractures. Technique and prospective results. Unfallchirurg.

